# Bond Wire Fatigue of Au, Cu, and PCC in Power LED Packages †

**DOI:** 10.3390/mi14112002

**Published:** 2023-10-28

**Authors:** Bernhard Czerny, Sebastian Schuh

**Affiliations:** Department Energy and Environment, Applied Electronics and Photonics, University of Applied Sciences Burgenland, Campus 1, 7000 Eisenstadt, Austria; sebastian.schuh@fh-burgenland.at

**Keywords:** accelerated mechanical fatigue testing, lifetime model, thermomechanical fatigue, CTE mismatch, physics of failure, frequency effect

## Abstract

Bond wire failure, primarily wire neck breakage, in power LED devices due to thermomechanical fatigue is one of the main reliability issues in power LED devices. Currently, the standard testing methods to evaluate the device’s lifetime involve time-consuming thermal cycling or thermal shock tests. While numerical or simulation methods are used as convenient and quick alternatives, obtaining data from material lifetime models with accurate reliability and without experimental fatigue has proven challenging. To address this issue, a mechanical fatigue testing system was developed with the purpose of inducing mechanical stresses in the critical region of the bond wire connection above the ball bond. The aim was to accelerate fatigue cracks at this bottleneck, inducing a similar failure mode as observed during thermal tests. Experimental investigations were conducted on Au, Cu, and Pd-coated Cu bonding wires, each with a diameter of 25 µm, using both low- and high-frequency excitation. The lifetime of the wire bond obtained from these tests ranged from 100 to 1,000,000 cycles. This proposed testing method offers material lifetime data in a significantly shorter timeframe and requires minimal sample preparation. Additionally, finite element simulations were performed to quantify the stresses at the wire neck, facilitating comparisons to conventional testing methods, fatigue test results under various operating conditions, material models, and design evaluations of the fine wire bond reliability in LED and microelectronic packages.

## 1. Introduction

**Figure 1:**
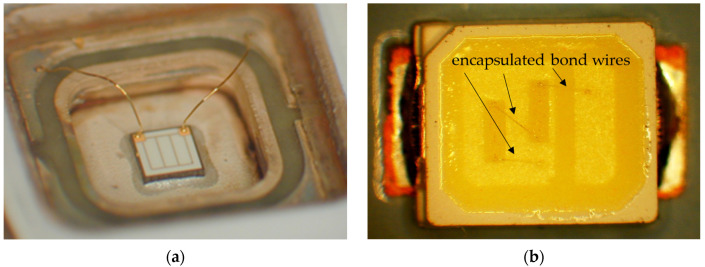
Bond wire connections in power LEDs; examples of LEDs (**a**) before encapsulation and (**b**) in an encapsulated package.

The rapidly increasing light-emitting diode (LED) market is continuously seeking out innovative design concepts to enhance device performance at a higher operation power and elevated temperatures and to reduce electrical losses in order to increase their reliability and lifetime [1,2]. However, the challenge lies in achieving cost reduction and a shorter time to market without compromising product quality. The LED and microelectronics packaging industries currently use both gold and copper wire bonding processes, with a growing preference for copper. Electrical connections between the semiconductor chip and the package are made using wire diameters ranging from 15 µm to 75 µm [3,4]. The choice of copper as a wire material, rather than gold, offers a number of benefits due to its material properties. These benefits include improved thermal and electrical properties, excellent ball neck strength, high loop stability, superior loop control, and reduced cost [4,5,6,7,8]. However, the material properties of copper with its higher yield strength than gold can result in higher stress and strain in the package and structure, or can lead to localized stress intensities. The inappropriate selection of copper wire bonding parameters can lead to reliability problems. Wire bonds are typically fabricated using thermosonic ball bonding technology. The process of wire bonding creates a heat-affected zone (HAZ) in the wire above the bonded area, affecting material properties through grain coarsening and playing a critical role in LED packaging’s reliability [9]. A power LED package and its wire bonds are shown in Figure 1 in a non-encapsulated and encapsulated state.

**Figure 2:**
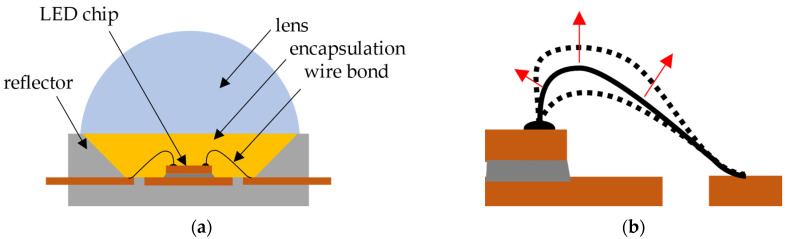
(**a**) Schematic structure of a power LED package and (**b**) the stress due to thermal expansion on the wire bond.

The primary concern regarding reliability, particularly for extended operations, is the thermal expansion and contraction of materials within the package during operation. A schematic structure of a power LED with its basic components and the effect of the thermal expansion of the package on the wire is illustrated in Figure 2. The wire bonds are subjected to repeated cyclical thermal and mechanical loading, resulting in the gradual accumulation of plastic strain, fatigue, and the eventual fracture of the wires. This weakening process acts as a critical link and can lead to device failure [10,11,12,13,14,15].

Cost-saving measures, such as dimming LEDs when full brightness is not needed, can cause efficiency problems and discomfort due to strong flickering effects at low brightness levels due to an inadequate selection of driver and dimming method. The occurring current ripples in such cases and the resulting cyclic thermomechanical stresses can lead to fatigue failure at critical interconnects in the LED package, such as wire bond connections, and thus cause a serious lifetime issue. In certain applications, such as the automotive industry, there is a requirement for fault-free performance for over a decade. It is therefore essential to characterize the fatigue properties over the low cycle fatigue (LCF) to high cycle fatigue (HCF) regimes, particularly in automotive and high-power electronics applications.

At present, LED package qualifications rely on time-consuming thermal cycling and thermal shock tests or simple extrapolations from the static material parameters obtained using bending, pull, or shear tests with finite element modeling (FEM) simulations [16,17,18,19,20,21,22,23]. Highly accelerated reliability tests, designed to replicate typical application conditions in an accelerated manner, provide essential parameters to improve material models for life prediction and FEM simulations. This enables reliability to be assessed within a reasonable timeframe [11,24,25].

To achieve this objective, a mechanical fatigue testing system was specifically designed to apply mechanical stresses to the critical region of the bond wire connection, which is the HAZ. This accelerated testing approach aims to expedite the occurrence of fatigue cracks at this crucial bottleneck. The first results using this method were published by Czerny and Schuh in 2023 [26]. The subject of this investigation was to experimentally determine the fatigue life of the fine wire bonds used in power LEDs as a valuable parameter for lifetime calculations, comparisons, and simulations. The aim was to bridge the gap from the LCF to HCF results with one testing setup and thus provide a fast alternative without the need for custom-made samples or intricate sample preparations. Following the experimental investigation, mechanical FEM simulations were conducted to quantify the stresses induced at the HAZ, which allows a comparison of the induced stresses of this excitation method and the oscillation amplitude, position, frequency, and geometry.

## 2. Methodology

The fatigue testing method used in this investigation is based on a mechanical accelerated lifetime test known as BAMFIT [27,28,29]. The BAMFIT tester was developed to obtain the fatigue life of wire bonding at the bonding interface and heel cracking in the high cycle fatigue regime, particularly for the heavy wire bonds used in high-power electronics. The primary objective is to induce fatigue failure similar to that experienced during thermal and power cycling tests. Induced fatigue failure is achieved by using a resonant tweezers tool to pinch the wedge or ball bond near the bond surface. The wire bond is then excited laterally to the surface in the direction of bonding. This mechanical action creates shear stresses in the interface, ultimately leading to lift-off failure. The tester utilizes ultrasonic oscillations operating at 60 kHz, enabling rapid testing with results even above 100,000 cycles. To investigate a wire break in the HAZ, this method was modified to excite the wire at a specific distance from the bonding surface using a delicate resonance tweezers tool and a gentle gripping force to hold the wire without causing damage. This testing approach necessitates obtaining a specimen before encapsulation to access and excite the wire itself.

**Figure 3:**
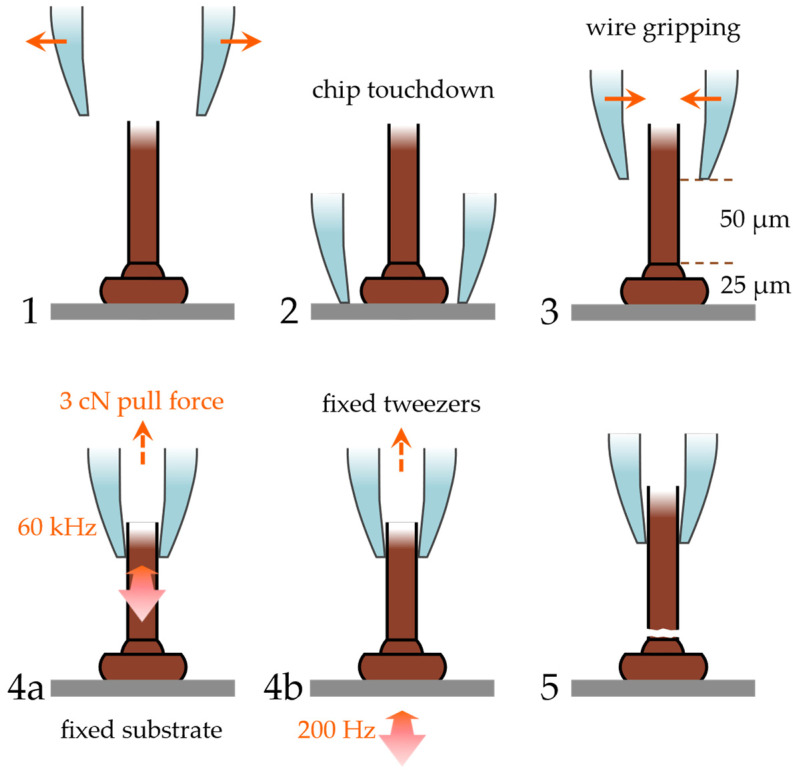
Fatigue testing procedure from (1–3) positioning and operation with (4a) oscillating tweezers at 60 kHz or (4b) oscillating stage at 200 Hz until (5) fatigue fracture. Reproduced with permission from Czerny, B.; Schuh, S [26].

The testing process of the fine wire is illustrated in Figure 3 and is conducted as follows: The resonance tweezers are aligned with the nailhead, in the direction of the bond wire, and then opened. They are then carefully lowered to the surface, with the movement measured using an inductance sensor in the tweezers’ suspension. Subsequently, the tweezers are raised to a height of 75 µm and then positioned to pinch the wire at a vertical distance of approximately 50 µm above the nailhead. At this particular position, the wire remains in a vertical orientation, disregarding the loop shape and length of the wire to the secondary bond. A slight vertical tension preload of 3 cN is applied and kept constant throughout the fatigue test. This preload serves to prevent buckling and also helps to lift the wire after a fracture occurs. The application of a differential displacement oscillation in the bonding direction of the tweezers and the chip stage induces the cyclic bending motion of the wire in the HAZ. This repeated bending action finally leads to a fatigue fracture at the neck above the nailhead.

**Figure 4:**
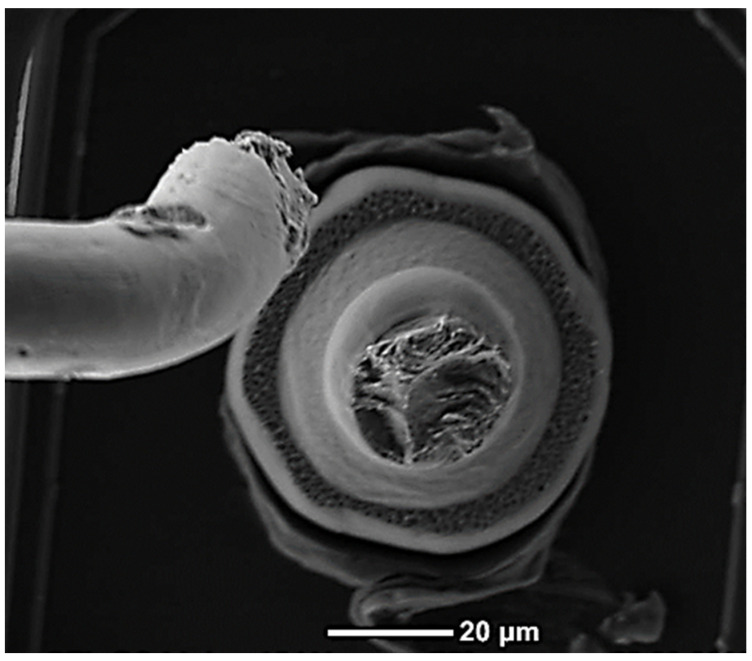
Fractured Cu wire in the HAZ above the nailhead after 200 cycles at 4 µm displacement amplitude at 200 Hz and the visible tweezers gripping marks on the wire above.

The test was conducted at two different frequency settings: first, at 60 kHz with the tweezers oscillating and the stage stationary, and second, at 200 Hz with the tweezers stationary and an oscillating shear piezo stage. The chosen frequency of 60 kHz is the operation frequency of the BAMFIT setup and 200 Hz is the optimal frequency of the shear piezo setup to reach up to 4 µm excitation amplitude. The reason for employing these two distinct excitation methods was that the stage, due to its mass, could not be accelerated while still maintaining the required displacement amplitudes. On the other hand, by operating the tweezers in resonance, it becomes feasible to achieve significant oscillations at a high frequency, but the same level of displacement is challenging to achieve when operating out of resonance at low frequencies. By utilizing both methods, it becomes possible to obtain lifetime data in both the HCF and LCF regimes within a reasonable timeframe. At each frequency, a specific oscillation amplitude was selected and calibrated using a laser Doppler vibrometer. For the 60 kHz setup, the calibrated oscillation amplitude at the tip of the tweezers tool was measured to be 75 nm. For the 200 Hz setup, two different oscillation amplitudes were determined: 1.3 µm at the edge of the bonding surface and 4 µm at the same location. Both excitation methods applied stresses to the wire, resulting in fatigue cracking directly above the nailhead within the HAZ, as depicted in Figure 4. To determine the number of loading cycles to failure (*N_f_*), the time of oscillation was measured until the inductive height sensor detected a sudden vertical movement at the point of complete fracture. The time measurement resolution is 15 ms, leading to an *N_f_* resolution of ±900 cycles for the tests at 60 kHz and ±3 cycles at 200 Hz. In both cases, this resolution is considerably smaller than the observed scattering of the obtained fatigue life data.

## 3. Experimental Results

The fatigue test was conducted on three different types of wires: Au, Cu, and Pd-coated Cu (PCC), each having a diameter of 25 µm. These wires were ultrasonically bonded to a contact pad on a chip using their respective optimized ball bonding tools and parameters. In the fatigue tests conducted, the main focus was on investigating the wire fatigue at the neck above the nailhead. Therefore, the shape of the wire and the length of the loop behind the tweezers position were not considered relevant to the specific analysis. Each wire material was tested using both the 200 Hz and 60 kHz setups, with a minimum of 10 samples tested for each material at each frequency. The maximum duration of a single test depended on the frequency and loading amplitude and was for the current settings less than 5 min until a complete fatigue fracture through the bond wire.

**Figure 5:**
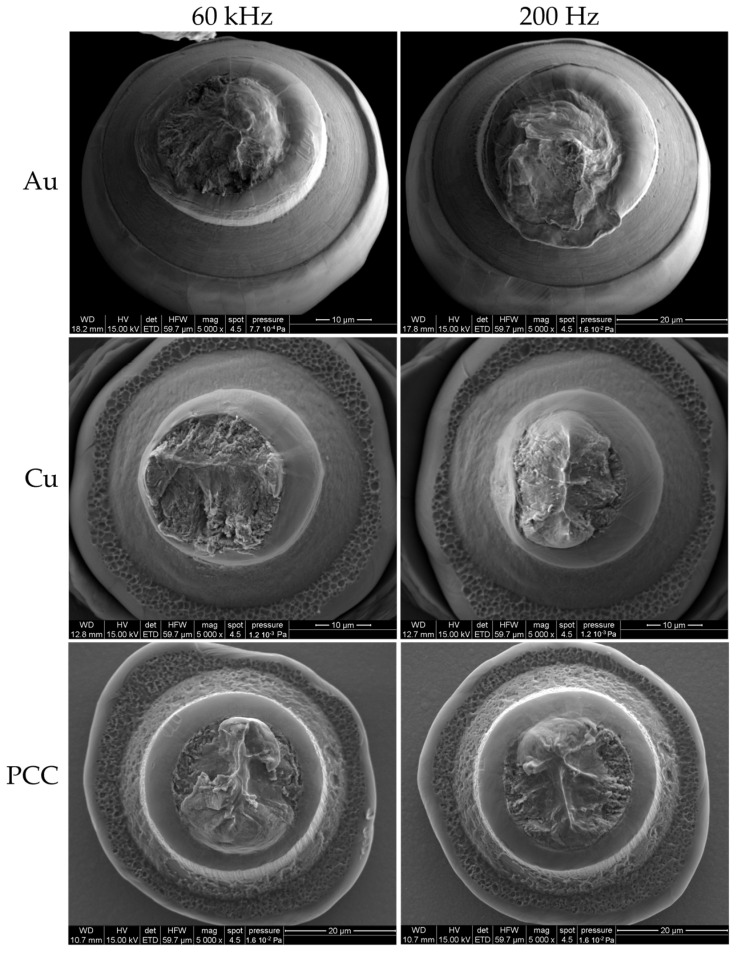
Wire bond fracture in the HAZ directly above the nailhead for Au, Cu, and PCC tested at 60 kHz and 200 Hz.

Figure 5 showcases a typical fracture surface for each wire material at both 200 Hz and 60 kHz. In all cases, the location of the fatigue fracture was observed at the neck above the nailhead, and there were no instances of fractures occurring at the bond interface or the tweezers gripping position for any of the materials tested. The fracture surfaces of the Au and Cu wires tested at 60 kHz exhibit distinct features indicative of high cycle fatigue, with fractures occurring perpendicular to the wire. For wires tested at 200 Hz, a different behavior is observed, showing much more ductile rupture and necking. PCC exhibits ductile and brittle fatigue characteristics in both LCF and HCF tests, distinguishing it from Au and Cu wires. In both testing modes, crack propagation starts with fine fatigue features perpendicular to the wire and progresses toward a large final ductile region. However, after testing at 200 Hz, the ductile region and necking become more pronounced. The significant contrast observed in the ductile and fatigue fracture surfaces between 200 Hz and 60 kHz is a result of the substantial difference in the excitation amplitude. Consequently, this leads to varying induced stresses per loading cycle.

**Figure 6:**
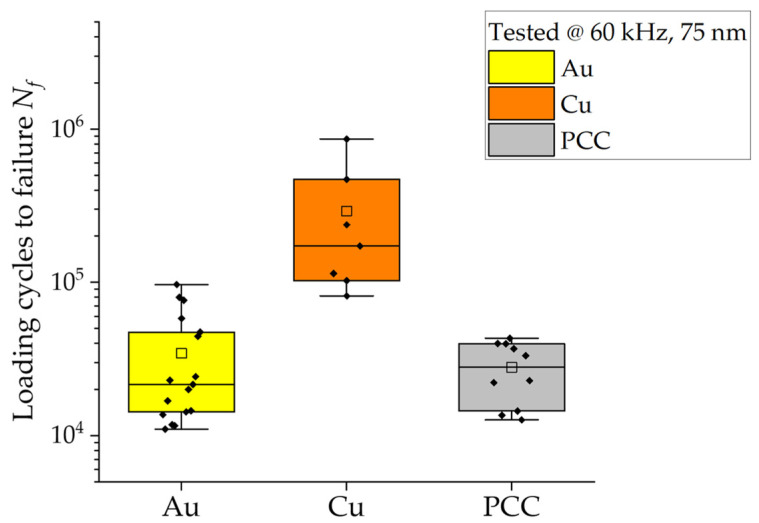
HCF lifetime results for Au, Cu, and PCC wires tested at 60 kHz and 75 nm displacement amplitude. Reproduced with permission from Czerny, B.; Schuh, S [26].

**Figure 7:**
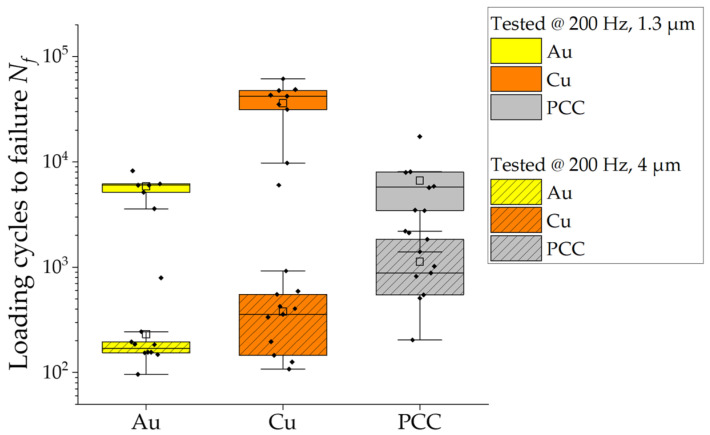
LCF lifetime results for Au, Cu, and PCC wires tested at 200 Hz and 1.3 µm and 4 µm displacement amplitudes.

Figure 6 presents a box plot diagram depicting the lifetime results for the Au, Cu, and PCC wires in the context of the HCF tests. Figure 7 displays the box plot diagram for the same wires in relation to the LCF tests. The results reveal a noticeable disparity between Au and Cu in the HCF tests at 60 kHz, with Au showing a lifespan approximately 10 times longer than Cu. PCC unexpectedly demonstrated comparable results to Au, achieving an average value of 30,000 cycles in the HCF tests. As for the LCF tests in Figure 7, the upper box plots above 2500 cycles were obtained with a lower loading amplitude of 1.3 µm. At a testing amplitude of 4 µm, Au shows the lowest fatigue life, with a small scattering at 100 to 300 cycles. Even though the mean value of Cu is above Au at 400 cycles due to the higher scatter, its lowest values are comparable to those for Au. The PCC with the highest lifetime of ~1000 cycles shows the highest *N_f_* results in contrast to the behavior at 1.3 µm amplitude and tests at 60 kHz. At 200 Hz and a displacement amplitude of 1.3 µm, Au and Cu behave in a similar way by increasing in *N_f_*, which for Cu, is up to seven times the lifetime of Au. The resulting fatigue life of PCC, on the other hand, does not behave in the same way as Au or Cu for a higher *N_f_*. It still reaches a higher fatigue life at a 1.3 µm excitation amplitude but does not nearly increase as much relative to Au or Cu. The Pd surface coating and/or microstructural differences seem to have a negative influence on the HCF but not as much on the LCF with large plastic deformations. This effect is not only seen at higher testing frequencies but also at lower amplitudes at 200 Hz, and the transition seems to be apparent already at 5000 loading cycles.

## 4. FEA Comparison

The experimental fatigue test conditions were further investigated using finite element analysis (FEA) to obtain the quantitative stress and strain values, allowing a comprehensive comparison of the fatigue stress levels and *N_f_* with other test methods or thermomechanical loading conditions commonly encountered in the field. The Au, Cu, and PCC wires were modeled in ANSYS with slightly different nailhead geometries according to the given shape of the nailheads. A multilinear kinematic hardening material model, obtained from annealed 25 µm Au and Cu investigations by Khatibi et al. [30] and PCC investigations, was extracted from tensile tests. A mesh size of <2 µm in the HAZ was used in this investigation with hexahedral meshing and continuous node sharing. The maximum von Mises stress values at the neck in the HAZ, where the fatigue crack starts to propagate through the wire, were used as the quantitative criteria for the comparison.

**Figure 8:**
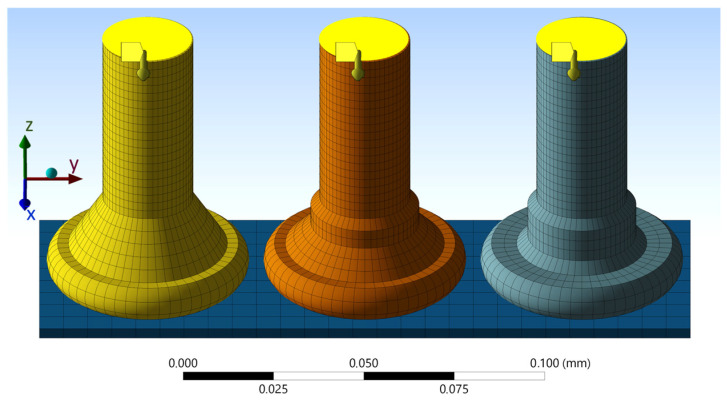
Au (**left**), Cu (**middle**), and PCC (**right**) wire bond models and meshes for static mechanical simulations of the fatigue test conditions.

A static mechanical simulation was conducted with a horizontal displacement of 75 nm, 1.3 µm, and 4 µm at the cross section of the wire at 75 µm above the chip surface, while restraining the degrees of freedom at the bottom of the Si chip, as displayed in Figure 8. At the beginning, the wire was considered to be in a stress-free state, and the wire loop behind the gripping tweezers was not simulated since the rest of the wire would not have an impact for this investigation.

**Figure 9:**
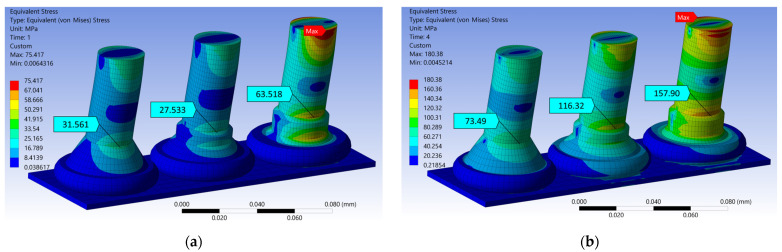
FEA results: von Mises stress for (**a**) 75 nm and (**b**) 4 µm displacement of the Au (left), Cu (middle), and PCC (right) wires.

**Figure 10:**
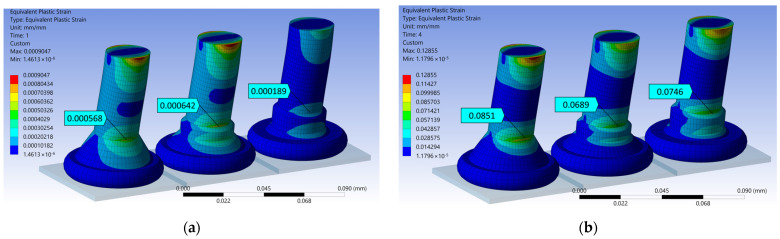
FEA results: equivalent plastic strain for (**a**) 75 nm and (**b**) 4 µm displacement of the Au (left), Cu (middle), and PCC (right) wires.

The calculated von Mises stresses are shown in the plots in Figure 9 for 75 nm and 4 µm displacement amplitudes, which resemble the excitation amplitudes at 60 kHz and 200 Hz, respectively. The equivalent plastic strain distributions for the same conditions are plotted in Figure 10. For every wire and displacement amplitude, the maximum stress and strain values were found at the neck region directly above the nailhead, aligned with the direction of the displacement. This area also corresponds to the location where the fatigue tests’ fracture surfaces exhibited signs of crack initiation. These maximum stress and strain values are given in Table 1 for each wire and displacement simulation, and also for the intermediate excitation amplitude of 1.3 µm.

## 5. Discussion

**Figure 11:**
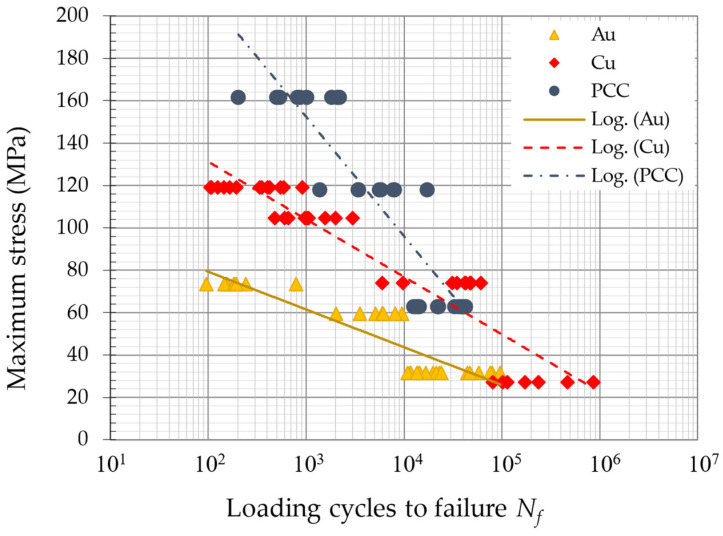
All experimental fatigue results plotted with the calculated maximum von Mises stress in the HAZ.

The FEA calculated stress and strain values for the presented fatigue test amplitudes allow a local stress level to be correlated with a quantitative lifetime for the investigated 25 µm bond wires. This correlation is with a good approximation independent of the loading conditions, such as bending, tensile compression, etc. Using this approach, all the test results can be plotted in a single diagram in Figure 11 with the maximum occurring stress, which is the local von Mises stress at the neck in the HAZ. In the case of this mechanical accelerated test, this is the same as the maximum stress amplitude for each cycle. In comparison to the box plot diagrams in Figure 6 and Figure 7, Figure 11 shows a direct lifetime comparison of the three wire materials, regardless of the testing frequency. The lifetime data on the *N_f_* of Au are 10 to 100 times lower than those of Cu or PCC, but the gap between Cu and Au decreases for a higher *N_f_*. The obtained fatigue data range between the LCF and HCF, and a linear fit in a single log plot is a simple way to describe the transition but is not sufficient for very low- and very high-fatigue prediction and does not include the testing frequency. The much higher stress levels in PCC compared to Cu and stress levels in Cu compared to Au are due to the relatively high yield strength of Cu and especially PCC.

In Equation (1), a four parameter lifetime model for the entire *N_f_* range was purposed by Weibull.

(1) $$S\left(N\right)=S_{d}+\frac{S_{max}-S_{d}}{\exp\left\{{\left(\frac{\log\left(N\right)}{\alpha }\right)}^{\beta }\right\}}$$

This relation describes the fatigue behavior using two fitting parameters *α* and *β*, which are determined to best fit the experimental fatigue data, the ultimate tensile strength (UTS) *S_max_*, and the endurance limit *S_d_*, and thus providing the fatigue stress *S* as a function of the loading cycles *N*.

Khatibi et al. [30] used this model to describe the behavior of the experimental fatigue results, which were conducted on 25 µm annealed coarse-grained Au and Cu wires in a LCF test under tensile–tensile loading (R = 0.11) at 0.05 Hz and in a HCF test under tensile–compression loading (R = 1) at 20 kHz. Due to the uniaxial applied loading forces, the stresses in the tensile investigations were experimentally determined using the measured applied forces or strain values against the wire diameter. Since the load ratio in the LCF at 0.05 Hz was under tension (R = 0.11), a Goodman conversion was used to calculate a stress amplitude for LCF and HCF comparison.

**Figure 12:**
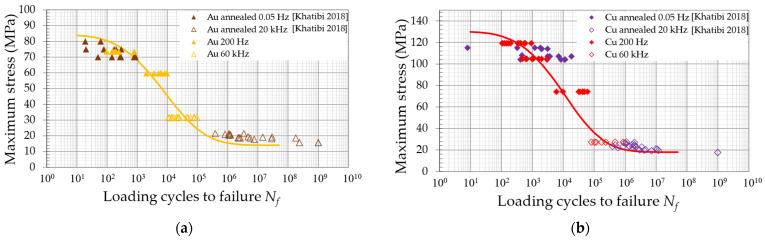
Comparison of the 200 Hz and 60 kHz bending fatigue results to uniaxial fatigue investigation by Khatibi et al. [30] of (**a**) annealed Au and (**b**) annealed Cu wires, each tested at 0.05 Hz (R = 0.11) and 20 kHz (R = 1).

For a direct comparison, the obtained *N_f_* at 200 Hz and 60 kHz can be compared to fatigue investigations by Khatibi et al. [30] for Au and Cu wires in Figure 12, by using the calculated maximum stresses (FEA) of the current bending fatigue and the experimentally determined total maximum stress values of the uniaxial fatigue tests. The lifetime results of both studies and the stress levels show similar results in the LCF and HCF regimes. This demonstrates that a simple static stress simulation of the test conditions with a well-known material model provides a good comparison of different loading conditions and fatigue experiments. The current investigation at 200 Hz and 60 kHz provides intermediate results in the transition between the LCF and HCF regimes.

**Figure 13:**
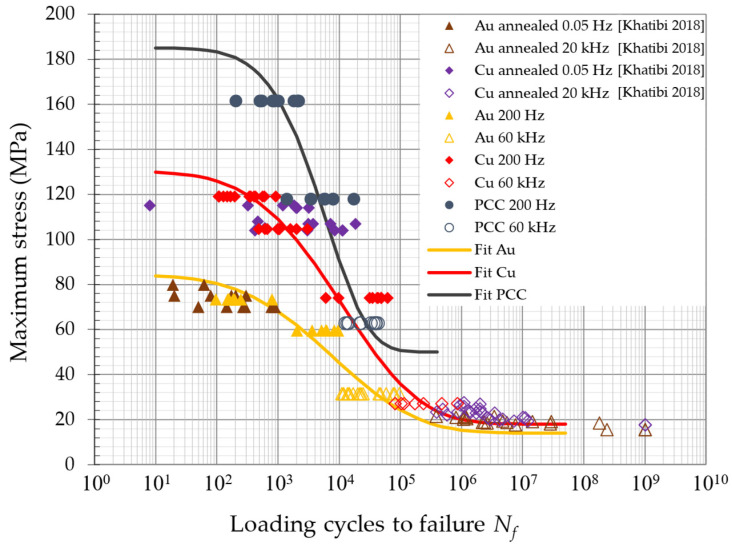
Au, Cu, and PCC fatigue data in comparison and the corresponding fitting curves optimized with the UTS and endurance limit in the lifetime model [30].

In Figure 13, all the obtained fatigue data on Au, Cu, and PCC and the literature’s data on the uniaxial fatigue test of Au and Cu wires are plotted on a single scale for comparison. The lifetime model in Equation (1) is included in Figure 12 and Figure 13 as fit curves. The parameters for the UTS and the endurance limit are taken from the experimental tensile tests and fatigue tests up until 109 loading cycles in [30]. The UTS for PCC was determined to be 185 MPa and the endurance limit estimated to be 50 MPa. The fitting parameters α and β were determined to fit the fatigue data obtained in the current investigation at 200 Hz and 60 kHz. All the parameters are given in Table 2. The fitting curves provide a good match to the fatigue data of both studies of Au and Cu. PCC with its high yield stress show a much steeper decrease in stress from LCF to HCF. The fatigue data of the current investigation provide a validation to the model, especially in the transition of LCF to HCF.

## 6. Conclusions

A versatile accelerated mechanical fatigue testing method to determine the lifetime of bond wires used in power LED packaging is proposed and evaluated in this study. The focus of the failure mode is bond wire breakage in the neck in the HAZ. Experimental investigations were conducted on Au, Cu, and PCC wire bonds prior to encapsulation using an accelerated mechanical fatigue test setup in both LCF and HCF regimes. The obtained lifetimes of the wire bonds ranged from 100 up to 1,000,000 cycles. Notably, the Au wire bonds exhibited the lowest lifetime among all test conditions, with a significantly higher difference in the LCF regime, being a factor of 10 lower than the Cu wires.

Using FEM simulations, the maximum stress values at the HAZ for the mechanical testing method were calculated, which provides comparison to other investigations and loading conditions. The results show a very good agreement of this bending fatigue with conventional fatigue tests of tension–tension and tension–compression loading of wire, despite the different testing conditions, used testing frequencies, and loading mode. This indicates that in the case of a similar investigated material and a well-known material model for FEA comparison, this fatigue testing approach provides a good alternative to conventional fatigue tests, which may be time-consuming or require intricate sample preparation.

The proposed accelerated mechanical fatigue testing method enables the acquisition of material lifetime data in a remarkably short timeframe and with minimal sample preparation. When combined with FEA, this method opens up various possibilities, such as comparative fatigue tests, assessments of material models, and design evaluations concerning the reliability of fine wire bonds in LED and microelectronic packages.

## Figures and Tables

**Table 1:** Static mechanical FEA results of fatigue test conditions.

	75 nm Displacement FEA (HCF @ 60 kHz)		1.3 µm Displacement FEA (LCF @ 200 Hz)		4 µm Displacement FEA (LCF @ 200 Hz)	
Wire	Max. von Mises Stress [MPa]	Equivalent Plastic Strain [mm/mm]	Max. von Mises Stress [MPa]	Equivalent Plastic Strain [mm/mm]	Max. von Mises Stress [MPa]	Equivalent Plastic Strain [mm/mm]
Au	31.561	5.679 × 10^−4^	57.818	3.057 × 10^−2^	73.49	8.51 × 10^−2^
Cu	27.533	6.422 × 10^−4^	73.48	2.456 × 10^−2^	116.32	6.896 × 10^−2^
PCC	63.518	1.888 × 10^−4^	116.01	2.654 × 10^−2^	157.90	7.46 × 10^−2^

**Table 2:** Material and fitting parameters for Au, Cu, and PCC used in the lifetime model.

Wire	UTS *S_max_* [MPa]	*S_d_* [Mpa]	α	β
PCC	185	101	8.956	6.535
Au	84	80	9.736	3.913
Cu	130	78	9.981	4.247
